# Personalization of pharmacotherapy with sirolimus based on volumetric absorptive microsampling (VAMS) in pediatric renal transplant recipients—from LC-MS/MS method validation to clinical application

**DOI:** 10.1007/s43440-024-00663-9

**Published:** 2024-10-10

**Authors:** Arkadiusz Kocur, Agnieszka Czajkowska, Kamila Rębis, Jacek Rubik, Mateusz Moczulski, Bartłomiej Kot, Maciej Sierakowski, Tomasz Pawiński

**Affiliations:** 1 Department of Drug Chemistry, Pharmaceutical and Biomedical Analysis, Faculty of Pharmacy, Medical University of Warsaw, Banacha 1, Warsaw, 02-097 Poland; 2 Therapeutic Drug Monitoring, Clinical Pharmacokinetics and Toxicology Laboratory Unit, Department of Clinical Biochemistry, The Children’s Memorial Health Institute, Dzieci Polskich 20, Warsaw, 04-730 Poland; 3 Department of Nephrology, Kidney Transplantation and Arterial Hypertension, The Children’s Memorial Health Institute, Dzieci Polskich 20, Warsaw, 04-730 Poland; 4 Institute of Biological Sciences, Cardinal Stefan Wyszynski University, Kazimierza Wóycickiego 1/3, Warsaw, 01-938 Poland

**Keywords:** Sirolimus, Rapamycin, VAMS, LC-MS/MS, Pediatrics, DLLME

## Abstract

**Background:**

The benefits of pharmacotherapy with sirolimus (SIR) in pediatric transplant recipients are well established. Traditionally, whole blood samples have been used to measure SIR concentrations. Volumetric Absorptive Microsampling (VAMS) is an alternative sampling strategy suitable for Therapeutic Drug Monitoring (TDM). In this study, we developed and validated two liquid chromatography-tandem mass spectrometry (LC-MS/MS) methods for determining SIR concentrations in whole blood (WB) and capillary whole blood samples collected using a VAMS-Mitra™ device.

**Methods:**

We used protein precipitation during WB sample preparation and dispersive liquid-liquid microextraction (DLLME) with methyl tert-butyl ether for VAMS sample preparation to optimise the analyte extraction process. The described validation protocols were cross-validated, confirming the equivalence of the whole-blood and VAMS-based methods. Furthermore, the developed methods were evaluated in two three-level rounds of an external proficiency-testing scheme.

**Results:**

The analytical methods were successfully validated within the calibration range of SIR (0.5–60 ng/ml). The validation parameters met the European Medicines Agency (EMA) and the International Association of Therapeutic Drug Monitoring and Clinical Toxicology (IATDM&CT) acceptance criteria. No hematocrit (tested in the range of 24.3–64.1%), matrix, or carry-over effects were observed. Cross-validation confirmed the interchangeability between VAMS-LC-MS/MS and WB-LC-MS/MS methods. The developed methods were successfully implemented for SIR determination in 140 clinical samples (70 each of WB and VAMS) from pediatric renal transplant recipients, demonstrating their practicality and reliability.

**Conclusion:**

The VAMS-based method has been rigorously tested and is clinically equivalent to the reference WB-LC-MS/MS method. Additionally, clinical validation confirmed the utility of the presented methods for TDM of the SIR in the pediatric population after renal transplantation.

**Graphical abstract:**

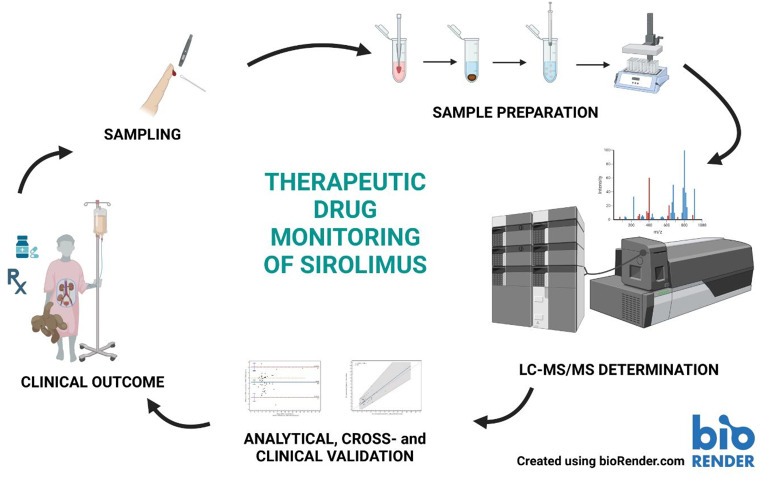

**Supplementary Information:**

The online version contains supplementary material available at 10.1007/s43440-024-00663-9.

## Introduction

Therapeutic drug monitoring (TDM) is a crucial aspect of pharmacotherapy that involves narrow therapeutic index drugs, particularly immunosuppressive agents. Sirolimus (previously known as rapamycin; SIR; Rapamune^®^) is a metabolic product of *Streptomyces hygroscopicus* and a mammalian target of rapamycin inhibitor [1]. Owing to the narrow therapeutic index, high inter- and intra-individual variability in drug pharmacokinetics, and potentially serious adverse events (including chronic and acute graft rejection), the dosage of SIR must be individualised to provide the best possible clinical outcomes [1, 2].

The SIR is routinely monitored in clinical practice by measuring the trough concentration parameter (C_0_), with typical concentrations ranging from 3 to 20 ng/mL in collected whole blood. SIR is highly bound to red blood cells (> 95%), making whole blood (WB) an appropriate matrix for drug determination [1, 2]. The distribution of SIR concentrations in whole blood is highly variable among renal transplant recipients, further emphasizing the importance of TDM. Clinical laboratories routinely use chromatographic methods and immunochemical assays (IAs) to determine SIR levels. Liquid chromatography-tandem mass spectrometry (LC-MS/MS) is a specific and selective technique considered the reference method for SIR determination. IAs are widely utilised in TDM laboratories because of their high automation, commercialisation, and ease of use [1]. Currently, Chemiluminescent Microparticle Immunoassay (CMIA), antibody-conjugated magnetic immunoassay (ACMIA), and Enzyme Multiplied Immunoassay Technique (EMIT) are used as IAs, with calibrations ranging from 2 to 30 ng/mL. A significant bias is observed in IAs compared to LC-MS/MS techniques due to cross-reactivity with drug metabolites. This may result in a 40% overestimation of SIR concentration [1–3].

Volumetric absorptive microsampling (VAMS) is an innovative blood collection method that encompasses both clinical biochemistry and TDM. This alternative approach reduces the amount of blood collected compared to traditional venipuncture and simplifies the pre-analytical process. One of the most widely used VAMS devices is the Mitra™ system (Trajan, formerly Neoteryx, Torrance, CA, USA), which offers various collection volumes ranging from 10 to 30 µl [6, 9–10]. According to the manufacturer, this technique is independent of the hematocrit effect, unlike the well-established dried blood spot (DBS) method [4]. VAMS also offers other advantages, including the ability for self-sampling by patients or guardians, ease of transport through shipping, and relatively high stability of most analytes. As an alternative to the classic DBS technique, devices other than Mitra™ might be applied. A quantitative dried blood spot (qDBS) device, namely Capitainer™, offers similar benefits to VAMS techniques, including hematocrit effect independence [9–10]. Several review articles have published more theoretical information on the use of VAMS and qDBS devices in laboratory and clinical practice [5–12].

The specificity of the pediatric population regarding pharmacodynamic and pharmacokinetic aspects of immunosuppressive drugs (including SIR) further justifies the benefits of individualization therapy using TDM– children are not small adults [1, 5]. This is due to the body’s physiological immaturity and factors related to drug interactions, such as affecting their metabolism [13]. More often than adults, pediatric patients require dosing changes, mainly due to intense growth and development. Additionally, non-compliance is more characteristic of children and adolescents than adults, directly affecting the effectiveness of therapy and the survival of the transplanted organ [5]. Therefore, microsampling techniques such as VAMS or qDBS devices seem to be a tool additionally adherence monitoring, including remote home-based self-sampling processes [5, 7–9].

This study aimed to develop, validate, and implement a novel analytical method that combines VAMS with liquid chromatography-tandem mass spectrometry (LC-MS/MS) for routine clinical practice in pediatric transplant centres. Additionally, this method has been successfully applied to a specific patient group, namely pediatric renal transplant recipients, thereby demonstrating its practical utility in real-world clinical settings.

## Materials and methods

### Reagents and materials

The standard reference of SIR (chemical purity ≥ 99.94%; Product No. HY-10219), its stable isotope-labelled internal standard (SIL-IS), SIR-d_3_ (95.48% chemical purity; Product No. HY-10219 S) were obtained from Med Chem Tronica (Sollentuna, Sweden). Ascomycin (ASC, chemical purity ≥ 95.00%; Product No. PA-03-1406-A) and temsirolimus (TEMS, chemical purity ≥ 98.00%; Product No. PZ0020) were delivered by Pol-Aura (Zawroty, Poland) and Sigma-Aldrich (St. Louis, MO, USA), respectively. Organic solvents for the mobile phase and rinse mixtures preparation (acetonitrile, Product No. 1.00029; methanol, Product No. 1.06035; and 2-propanol, Product No. 1.02781, all with LC/MS-grade) were acquired from Merck (Darmstadt, Germany). Eluent additive modificators, such as ammonium formate (> 99.99% chemical purity; Product No. 70221) and formic acid for LC-MS (> 99.99% chemical purity; Product No. 5.33002), were bought from Supelco (Bellefonte, PA, USA). Copper(II) sulfate pentahydrate (CuSO_4_·5H_2_O, ≥ 99.00% purity; Product No. C7631) was purchased from Sigma-Aldrich (St. Louis, MO, USA). Methyl tert-butyl ether (MTBE; Product No. 9042-02), LC grade, was obtained from J.T. Baker (Deventer, the Netherlands). Deionised-grade reagent-water was produced using a DL2-400 system (Polwater, Kraków, Poland). Blank whole blood (WB) samples anticoagulated with K_3_-EDTA, but without analyte, were obtained from healthy donors at the Regional Centre of Blood Donation and Hemotherapy (Warsaw, Poland). WB samples were stored at 4 °C for no longer than one week.

S-Monovette^®^ test tubes (1.2 mL) containing K_3_-EDTA as an anticoagulant for whole-blood collection, lancets, and blood collection sets were acquired from Sarstedt (Nümbrecht, Germany). VAMS-Mitra™ microsampling devices for 10 µL of capillary whole blood collection in clamshell racks for sampler drying were purchased from Trajan (formerly Neoteryx, Torrance, CA, USA).

Plastics, laboratory pipette tips, and test and falcon tubes were obtained from GenoPlast Biotech (Rokocin, Poland). Eppendorf Reference 2 (Eppendorf, Hamburg, Germany) automatic pipettes were used for stock, working, calibrators and quality control solutions preparing, and clinical samples as well. Glass chromatographic vials, 300 µL poly-spring glass inserts, and complementary polytetrafluoroethylene screw caps were obtained from Alwsci Technologies (Zhejiang, China).

### Stock, working and calibration solutions

Primary stock solutions were prepared by dissolving a weighed quantity (10 mg) portion of the solid standard (using MX analytical balance with ± 0.01 mg precision, from Mettler-Toledo, Columbus, OH, USA) in a methanol-water mixture (1:1, v/v) to obtain 1 mg/mL of SIR, SIR-d_3_, ASC, and TEMS solutions (the sonication was applicated for solubility improvement when necessary). The solutions were prepared in a Duran glass volumetric flasks characterized by A class (Brand GmbH, Wertheim, Germany). Subsequently, the primary SIR, SIR-d_3_, ASC, and TEMS solution was appropriately diluted using a methanol-water mixture (1:1, v/v) to obtain 10,000, 1000 and 100 ng/mL working intermediate solutions. SIR solutions generated working calibration standards and working quality control standards by adequate dilution (details in Supplementary file) with pure deionized water. The procedure was duplicated for SIR, following EMA guidance according to the necessity of preparing working calibration standards and working calibration controls using two independent stock solutions [14]. Fixed amounts of the SIR-d_3_, ASC, and TEMS diluted stock solutions were mixed with pure deionized water to obtain each IS’s 125 ng/mL concentration level. Stock and working solutions of SIR, SIR-d_3_, ASC, and TEMS have been stored at -40 °C, portioned in 2-mL glass vials, no longer than 6 months.

The main calibration for WB-LC-MS/MS quantification of SIR was carried out according to the following protocol. During the validation of the analytical method for the determination of SIR in WB (reference method), calibrators (CS) were prepared by spiking 35µL of WB with 3.5µL of corresponding SIR working calibration solution to obtain appropriate analyte concentrations of 0.25, 0.5, 1.0, 2.5, 5.0, 10.0, 30.0, and 60.0 ng/mL. QC samples were prepared using the same procedure at four different levels: 0.35 ng/mL (lower - LQC), 0.75 ng/mL (1st medium– MQC_1_), 3.50 ng/mL (2nd medium– MQC_2_), 25.0 ng/mL (3rd medium– MQC_3_), and 45.0 ng/mL (higher - HQC). A zero sample (without analytes and ISs) and a blank sample without SIR were prepared using the same procedure with pure water as a CS and/or IS surrogate. CS, QC, blank and zero samples were prepared freshly for each clinical sample set.

Mitra™ VAMS calibration samples were prepared by gently loading of 10 µL of whole blood spiked with corresponding CS or QC. During the VAMS-LC-MS/MS method validation, the CS was characterized by the same nominal concentrations: 0.25, 0.5, 1.0, 2.5, 5.0, 10.0, 30.0, 60.0 ng/mL. Similarly to above, the concentration of QC was set as follows: 0.35 ng/mL (lower - LQC), 0.75 ng/mL (1st medium– MQC_1_), 3.50 ng/mL (2nd medium– MQC_2_), 25.0 ng/mL (3rd medium– MQC_3_), and 45.0 ng/mL (higher - HQC).

Preparation protocol of working CS and working QC is provided as Supplementary File (part 1).

### Chromatographic and mass spectrometry conditions (LC-MS/MS)

Analyses were carried out using an ultra-high-performance chromatographic system, the Nexera X2 (Shimadzu, Tokyo, Japan), which consisted of a pump with a mobile phase mixer (LC-30AD), degasser unit (DGU-20A5R), autosampler device with a thermostat (SIL-30AC), and thermostatic column (CTO-20AC) connected to an LCMS-8050 triple quadrupole MS detector (Shimadzu, Tokyo, Japan). The Poroshell 120 EC-C18 column, 50 × 4.6 mm, 2.7 μm (Agilent Technologies, Santa Clara, CA, USA), guarded by a Poroshell 120 EC-C18 pre-column, 5 × 4.6 mm, 2.7 μm (Agilent Technologies, Santa Clara, CA, USA), was maintained at 60 °C in the oven. The mobile phase comprised of two solutions employed in the gradient flow mode at a flow rate of 1 mL/min. The first solution (phase A) was water containing ammonium formate (2 mM) and 0.1% MS-grade formic acid, whereas the second solution (phase B) was methanol with ammonium formate (2 mM) and 0.1% MS-grade formic acid. The phases were stored at 4 °C in the fridge, and the estimated expiration date was set as a minimum of one week (the solutions have been completely worn out during this period). The gradient program began with 95% phase A and 5% phase B for the initial 0.49 min, followed by a change to 5% phase A and 95% phase B between 0.50 and 2.49 min. At 2.50 min, the mobile phase returned to the initial conditions. The total run time was 3.50 min, with 5 µL and 1 µL injection volumes for WB- and VAMS-based methods, respectively.

Mass spectrometry analysis was performed using electrospray ionisation (ESI) in positive mode with multiple reaction monitoring (MRM). The ammonium adduct of SIR, [M + NH_4_]^+^, was monitored using the following transitions: 931.70→882.45 (quantitative pair) with a collision energy (CE) set at -12 eV, and 931.70→864.35 (qualitative pair) with CE set at 15 eV. MRM pairs for the internal standards (ISs) were also established: 934.60→864.50 (for d_3_-SIR; CE = -12 eV), 809.55→756.35 (for ASC; CE = -21 eV), and 1047.80→980.40 (for TEMS; CE = -20 eV). The dwell time for all the monitored MRM pairs was fixed at 8 ms. The MS apparatus parameters were optimised using LabSolutions 5.97 software (Shimadzu, Tokyo, Japan) at the following values: interface temperature, 200 °C; desolvatation temperature, 355 °C; temperature of the desolvatation line, 200 °C; and heat-block temperature, 400 °C. Nitrogen was employed as the drying gas and nebulizing gas at flows of 3 L/min and 2 L/min, respectively. Compressed air was utilised as the heating gas with a 17 L/min flow, whereas argon was used as the collision-induced dissociation gas at a pressure of 270 kPa. The needle was rinsed before and after aspiration using a methanol: acetonitrile: 2-propanol: water (1:1:1:1; v/v/v/v) mixture in the internal and external purging modes to eliminate the carry-over effect. Samples were stored in an autosampler at 5 °C.

LabSolutions software version 5.97 (Shimadzu, Tokyo, Japan) was used for peak marking, counting, and calibration using 1/x weighting and signal-to-noise (S/N) evaluation.

### Protocol of sampling

1.2 mL of WB for the K_3_-EDTA test was collected using the classic venipuncture method, whereas 10 µL of capillary whole blood was collected simultaneously using a Mitra™ tip. A total of 140 samples (70 VAMS and WB each) were collected from pediatric renal transplant patients (*n* = 25) during routine follow-up visits to the Renal Transplant Outcome Clinic at the Children’s Memorial Health Institute (Warsaw, Poland) between May 2023 and February 2024. All patients received Rapamune^®^ (Pfizer, NY, USA) in a personalized therapeutic regimen as an oral solution or tablet, concomitantly with other immunosuppressants. The patient’s demographic and clinical data included in the study are presented in Table 1. All samples were collected before the first daily dose of the drug for trough concentration measurements using WB-LC-MS/MS and VAMS-LC-MS/MS assays. The WB samples were stored at 4 °C for one week, whereas the VAMS tips were stored at ambient temperature, with desiccant in the dark, for 2 weeks until LC-MS/MS analysis.

**Table 1:** Summarized demographic data and clinical results of sirolimus determination in pediatric patients recruited to study. Data are presented as mean with standard deviation (mean ± SD) and range (min - max). The values are expressed with corresponding units indicated with variable names. Abbreviations: SIR, sirolimus

Variable	Patients’ data
number of patients	25
total number of samples	140 (70 samples for each method)
sex [male/female]	13/12
age [years]	13.46 ± 2.99 (4.23–17.92)
body weight [kg]	33.56 ± 13.27 (12.24–66.23)
height [m]	1.50 ± 0.21 (0.88–1.68)
SIR dosage form [solution/tablets]	8/17
SIR daily dose [mg]	3.54 ± 0.50 (0.6–4.5)

All patients and their legal guardians provided written informed consent before being included in the study. The study was conducted in accordance with the Declaration of Helsinki, Council for International Organizations of Medical Sciences Guidelines, and Good Clinical Practice. The project was approved by the Bioethics Committee of the Children’s Memorial Health Institute in Warsaw (number of agreements: 15/KBE/2023 with later amendments).

### Analytical samples preparation protocol and methodology

The WB-LC-MS/MS assay clinical sample preparation protocol included 35µL of whole blood diluted with an equal amount of pure deionized water for hemolysis initiation. In the case of spiked samples for methods validation, calibrators and QC preparation, to above mentioned whole blood volume, 3.5 µL of corresponding working solution and 31.50 µL of deionized water were added. Next, the sample was enriched with internal standards mixture solution (the final concentration of each IS was 15 ng/mL). Protein precipitation was initiated using a mixture of 250 µL of 0.1 M CuSO_4_:ACN (1:1, v/v). Subsequently, the obtained mixture was vortexed for 1 min at 1000 RPM using IKA vortex (IKA, Warsaw, Poland) and incubated at -40 °C in an ice bath. The supernatant was obtained using centrifugation (for 5 min., at 0 °C, 2138×g) with fixed-rotor MPW-375 centrifuge, and finally, 200 µL of them was transferred into the chromatographic vial. The 1µL of the obtained solution was injected into the LC-MS/MS platform.

VAMS-Mitra™ samples were collected following the manufacturer’s instructions [15]. For calibrator, as well as QCs preparation during VAMS-LC-MS/MS method validation, the 10 µL of blood enriched corresponding working solution (35 µL of blood spiked with 3.5 µL of SIR working solution in polypropylene test tube) of SIR was gently absorbed using Mitra™ tip. In both scenarios, the sampler with collected blood was dried at room temperature for at least two hours in either clamshells or auto-racks with desiccant (silica gel bag). The VAMS Mitra™ sampler tip was transferred to a polypropylene tube and was extracted using 150 µL of pure deionized water. The extraction process was supported using sonification (15 min., 40 °C) and subsequently shaking using a thermoblock vortex (Thermo Scientific, Waltham, MA, USA) at RT for 15 min. (1000 rpm). After that, the sample was spiked with ASC (internal standard, final concentration 15 ng/mL) and purified using 150µL of precipitation mixture (the same ingredients as in the WB sample pretreatment protocol). Then, the sample was treated similarly to the WB-LC-MS/MS preparation protocol. 200µL of the obtained supernatant has undergone an additional step. The 1 mL of MTBE/ACN mixture (1:1), v/v) was dispersed into the sample using a Hamilton syringe delivered by Phenomenex (Torrance, CA, USA). Next, phase separation was supported using sample incubation at -40 °C, and the obtained organic phase was transferred into a glass Pyrex tube (Corning, NY, USA) and evaporated under a nitrogen stream to dryness (at 40 °C). The residue was reconstituted with 150µL of mobile phases mixture (A: B, 1:1, v/v) and transferred to a chromatographic vial with insert. The 5µL of solution was injected into the LC-MS/MS system.

### Method validation

In this study, the guidelines set forth by the European Medicines Agency (EMA) and/or the Food and Drug Administration (FDA) were employed for bioanalytical method validation [14, 16]. Moreover, the International Association of Therapeutic Monitoring and Clinical Toxicology (IATDM&CT) guidelines have been implemented for analytical method validation [17, 18].

During the development of this method, three internal standards (IS) were used to validate the WB-LC-MS/MS method. The EMA guidelines recommend using a stable isotope-labelled IS (SIL-IS) when its isotopic purity is deemed satisfactory, but in this case (besides d_3_-SIR), structural analogues of SIR—ASC and TEMS were also used [14].

The following parameters were evaluated in both the WB-LC-MS/MS and VAMS-LC-MS/MS methods: selectivity, specificity, lower limit of quantification (LLOQ), calibration and linearity, accuracy, imprecision, carry-over, matrix effect, short- and long-term stability, and incurred sample reanalysis (ISR). Additionally, the hematocrit-dependent recovery of analytes from the VAMS samples was tested.

Blank (without IS, without SIR) and zero samples (without analytes) were analysed for selectivity using WB and VAMS samples from eight healthy volunteers. The following guidelines consider the selectivity satisfactory if the detector response to interfering components is lower than 20% for the analyte (LLOQ) and 5% for the IS [14, 16]. The specificity of this method was evaluated similarly. Calibration and linearity were assessed using the 1/x weighting method, with a calibration range of 0.25-60.0 ng/mL and 15 calibration curves were evaluated for each method during validation. Accuracy and imprecision CV were assessed within and between ten repetitions for both methods, with acceptance criteria requiring mean values within 15% of the reference value and LLOQ within 20%. According to the IATDM&CT guidelines, the mean CV% should be less than 10% (or even ≤ 6%). The carry-over effect was evaluated in 15 different runs using a protocol based on injecting the HQC sample (60 ng/mL) immediately before injecting the double-blank sample. The carry-over effect is defined as the response of the analyte and IS in the blank sample lower than 20% and 5% of the response at the LLOQ, respectively [14, 16]. The characteristics of the LC-MS/MS methods, including the matrix effect (ME), process efficacy (PE), and absolute recovery (AR) percentage ratios, were evaluated at two QC levels (LQC and HQC) using six blank samples from healthy donors, following a protocol published by Taylor [19]. This parameter’s acceptable accuracy and precision should be within ± 15% of the nominal concentration or not higher than 15% [14, 19].

The stability evaluation included the performance of various experiments, such as freeze-thaw stability and sample stability in an autosampler as well as the long-term stability of the VAMS tips under different stress conditions. The stability was considered acceptable if the differences were within the nominal range of ± 15% [14, 16].

According to EMA guidelines, ISR experiments should be performed considering the presence of metabolites, concomitant drugs, and non-homogeneity in the analyzed sample. The experiment involved repeating the assay for 10% of the samples included in the study and calculating the mentioned parameter description as the ratio of differences between results to the mean value of the measurements. The accepted criterion is that at least 67% of the repetitions must meet the acceptance criterion of 20% mean difference [14, 16].

Following recommendations, the potential hematocrit effect should be evaluated at three hematocrit levels for two different QC samples in six repetitions (through assessment of recovery rate regarding hematocrit value) as well as during clinical validation (through evaluation of the relationship between individual hematocrit value and the difference between analyte levels in whole blood and VAMS sample) [18]. The Passing-Bablok regression calculation, Bland–Altman bias estimation and correlation methods are routinely used to evaluate paired results and assess the methods’ equivalence. Acceptance criteria for the Bland-Altman approach demand that the calculated mean bias be less than 20% for at least 67% of the paired samples examined [20, 21]. According to the IATDM&CT guidelines, the novel analytical technique (compared with the reference method during regression evaluation) should fulfil the following criteria: the slope should be within ± 10% of the theoretical value of 1.0, and the intercept should not exhibit a statistically significant difference from zero [17]. The mean bias calculated for paired samples should be lower than 15% for 67% of the analysed samples [17].

### Statistical analysis

MedCalc software version 22.023 (MedCalc Software Ltd, Ostend, Belgium) was used for the statistical evaluation of the validation results, including cross- and clinical validation (Passing–Bablok regression and bias estimation using the Bland–Altman methodology for WB- and VAMS-LC-MS/MS methods comparison). Correlation tools, such as Pearson’s, Sperman’s rho and Intraclass correlation coefficient, were used for the Results of validation are presented as the arithmetic mean ± standard deviation (SD). In contrast, the coefficient of variation (CV) is presented as the SD/arithmetic mean percentage ratio. The Shapiro-Wilk test was used for the normality determination of the obtained data. In all performed statistical analyses, the p-value threshold equal to 0.05 was considered significant.

## Results

### WB-LC-MS/MS and VAMS-LC-MS/MS methods analytical development

Two LC-MS/MS methods have been developed for SIR determination using WB and VAMS. The chromatographic separation and MS parameters were adjusted experimentally based on the chemical properties of the analytes, and chromatograms were obtained (representative chromatograms are presented in Supplementary File - part 2). The SIR determination in WB was based on three internal standards: SIL-IS (RAP-d_3_) and structural analogues ASC and TEMS. After validation and statistical evaluation, ASC was determined to be the most suitable internal standard for the VAMS-LC-MS/MS method validation.

The sample preparation methodology was experimentally optimised. In the case of the WB-LC-MS/MS assay, one-step purification was assessed with a 0.1 M CuSO_4_: ACN (1:1, v/v) mixture. An additional step using the dispersive liquid-liquid microextraction (DLLME) is employed to improve the sensitivity of the VAMS-based method. MTBE was chosen as the preferred solvent because of its high recovery, which was supported by its high SIR chromatographic peak intensity. The retention time of SIR was 2.61 ± 0.11 min. (CV = 1.86%, *n* = 50), while for the internal standards, it was set as 2.51 ± 0.09 min. (CV = 0.99%, *n* = 50), 2.49 ± 0.23 min. (CV = 2.67%, *n* = 50) and 2.62 ± 0.19 min. (CV = 1.34%, *n* = 50) for ASC, TEMS, and RAP-d_3_, respectively. These statistical data show that the mobile phase provides reliable retention times. No interference was observed in the retention times. The total runtime of the analysis was 4.50 min.

### WB-LC-MS/MS method validation

The WB-LC-MS/MS method has been validated in the 0.25–60 ng/mL calibration range using ASC, TEMS, and SIR-d_3_ as the internal standard (IS). No interference from endogenous substances was observed during the chromatographic run, and the limit of detection (LOD) was found to be 0.10 ng/mL (S/*N* > 10). Extracted ion chromatograms analysed qualitatively for the interference from unknown or endogenous substances in the matrix during each analytical round. The linearity and calibration were assessed using 15 calibration curves, with average R^2^ values of 0.9996 ± 0.0011, 0.9989 ± 0.0021, and 0.9818 ± 0.0087 for calibration using ASC, SIR-d_3_, and TEMS, respectively. The average calibration equations were y = 0.4254x + 0.0050 for ASC, y = 0.3578x + 0.0099 for SIR-d_3_, and y = 0.2931x + 0.0983 for TEMS. The imprecision CV and accuracy validation parameters are listed in the Supplementary File (part 3). In the case of the validation process using ASC and SIR-d_3_, the acceptance criteria were fulfilled, with an accuracy within the range of 85–115%, imprecision of less than 15%, and less than 20% for LQC, and a CV of less than 10% [14, 16–17].

The carry-over effect was found to be insignificant for the SIR and IS standards and fulfilled the EMA criteria (0.643 ± 0.120% for SIR and 0.134 ± 0.088%, 0.112 ± 0.034%, and 0.196 ± 0.110% for ASC, SIR-d_3_, and TEMS, respectively). The WB samples were stable at 5 °C in the autosampler (*n* = 6; initial days 1–3–5 and after the 7-day checkpoint), fulfilled the acceptance criteria, were satisfactory, and were stable both before and after extraction [*n* = 6; for LQC and HQC]. The results of these experiments are listed in the Supplementary File (part 3). Moreover, the samples, working solutions, and calibrators were stable during at least three freeze-thaw cycles (data not shown).

The ME, AR, and PE were determined following pre- and post-extraction experiments to evaluate the matrix effect. These parameters illustrate the matrix effects in WB, and PL fulfils the EMA acceptance criteria. The calculated parameters are listed in Supplementary File (part 3).

For the ISR experiment, the percentage difference between pairs of samples was less than 20% for 80% of the samples (mean: -7.42 ± 2.75), which satisfied the EMA acceptance criteria.

### VAMS-LC-MS/MS method validation

The VAMS-LC-MS/MS method was validated in the 0.25–60 ng/mL calibration range using ASC as the internal standard (IS). The linearity parameter was evaluated based on fifteen repetitions of calibration curves. No endogenous substances interfered during the chromatographic run, and the limit of detection (LOD) was set as 0.10 ng/mL (S/*N* > 10). Extracted ion chromatograms were analyzed for possibility of interferences caused by unknown or endogenous substances from the matrix. Linearity and calibration were assessed based on 15 calibration curves (SIR to IS peak area about the standard concentration), with an average R^2^ of 0.9966 ± 0.0019 and an average calibration equation of y = 0.3966x − 0.0261. The imprecision CV and accuracy results are presented in Table 2, and the acceptance criteria were fulfilled (accuracy within 85–115%, imprecision less than 15% and less than 20% for LQC) [14, 16–17].

**Table 2:** Accuracy and imprecision CV parameters based on within-day (intra-day) and between-day (inter-day) repetition analysis for the VAMS-LC-MS/MS method [*n* = 10]. ASC, ascomycin; LOQ, lower quality control; MQC_1_– 1st medium quality control; MQC_2_– 2nd medium quality control; MQC_3_– 3rd medium quality control; HQC, higher quality control. Data are presented as mean with standard deviation (mean ± SD)

	LOQ 0.35 ng/mL	MQC_1_ 0.75 ng/mL	MQC_2_ 3.50 ng/mL	MQC_3_ 25 ng/mL	HQC 45 ng/mL
**IS: ASC** (within-day (intra-day))					
Mean concentration [ng/mL]	0.36 ± 0.03	0.79 ± 0.04	3.59 ± 0.16	25.34 ± 0.71	45.46 ± 0.86
Accuracy [%]	102.82	101.95	102.61	98.51	100.08
Imprecision (CV) [%]	7.08	5.29	4.53	2.81	1.88
**IS: ASC** (between-day (inter-day))					
Mean concentration [ng/mL]	0.35 ± 0.02	0.75 ± 0.02	3.51 ± 0.08	25.31 ± 0.41	45.27 ± 0.71
Accuracy [%]	96.20	98.35	99.15	101.12	99.72
Imprecision (CV) [%]	4.54	2.43	2.32	1.63	1.56

The carry-over effect was insignificant for SIR and ASC, with values of 0.522 ± 0.091% and 0.093 ± 0.071%, respectively. These results meet the EMA criteria [14].

The stability of the VAMS samples stored at 5 °C in the autosampler for LQC and HQC was evaluated using six samples, with initial measurements taken on days 1, 3, and 5, and a 7-day checkpoint. The results were deemed satisfactory (Table 3).

**Table 3:** Stability evaluation in the autosampler for the VAMS-LC-MS/MS method [*n* = 6]. ASC, ascomycin; LOQ, lower quality control; HQC, higher quality control. Data are presented as mean with standard deviation (mean ± SD)

Calculated concentration (ng/mL) and stability (%)					
	Initial	Day 1	Day 3	Day 5	Day 7
**IS: ASC**					
LQC– 0.35 ng/mL	0.37 ± 0.09; 100%	0.34 ± 0.14; 91.89%	0.33 ± 0.06; 90.12%	0.34 ± 0.05; 91.89%	0.32 ± 0.03; 86.49%
HQC– 45 ng/mL	44.83 ± 0.95; 100%	45.02 ± 1.18; 100.42%	44.12 ± 1.61; 98.42%	43.91 ± 1.18; 97.95%	43.55 ± 1.50; 97.17%

Furthermore, the samples, working solutions, and calibrators were stable after at least three freeze-thaw cycles. The VAMS samplers after collection were stable for one month, with storage temperatures of -20 °C (98.43 ± 1.33%), RT (in the dark; 90.15 ± 1.67%), and 4 °C (95.33 ± 2.01%). However, stability at 60 °C was only acceptable for three days (85.43 ± 2.17%).

The parameters mentioned above (ME, AR, and PE) were calculated during the matrix effect evaluation after the pre- and post-extraction experiments. The above parameters characterise the matrix effects in the WB, and the VAMS fulfils the EMA acceptance criteria [13, 14]. The data are presented in Supplementary material (part 3) and Table 4, respectively.

**Table 4:** Matrix effect (ME), absolute recovery (AR), and process efficiency (PE) summary for VAMS-LC-MS/MS using ASC as IS [*n* = 6]. ASC, ascomycin; LOQ, lower quality control; HQC, higher quality control. Data are presented as mean with standard deviation (mean ± SD). F represents IS normalized matrix factor

Parameter	LQC– 0.35 ng/mL			HQC– 45 ng/mL		
	SIR	IS-ASC	F ratio (SIR/ASC)	SIR	IS-ASC	F ratio (SIR/ASC)
ME	−18.99 ± 2.3%	−11.11 ± 2.01	101.36 ± 11.01	−12.32 ± 1.98	−9.95 ± 1.77	99.02 ± 6.33
PE	73.99 ± 2.85	69.69 ± 3.44	98.91 ± 1.77	71.98 ± 2.51	64.24 ± 4.04	102.31 ± 5.10
AR [%]	74.21 ± 3.01	71.09 ± 2.97	99.63 ± 1.99	75.11 ± 4.12	72.12 ± 2.08	98.99 ± 2.74

The findings of the ISR experiment were deemed to be satisfactory. The variation between sample pairs was less than 20% for 80% of the samples (mean: -6.33 ± 3.47), fulfilling the EMA acceptance criteria [13, 14].

Statistical analysis revealed no significant association between the differences in SIR levels (WB and VAMS) and individual hematocrit levels (Table 5). The HE was assessed in all the patients included in the study. (*p* = 0.0621; Spearman’s rho correlation coefficient: 0.2740; -0.0140–0.5200, 95% CI).

**Table 5:** Hematocrit effect evaluation on three hematocrit levels for two QCs– LQC and HQC measured as recovery rate (%) [*n* = 4]. LOQ, lower quality control; HQC, higher quality control. Data are presented as mean with standard deviation (mean ± SD)

Hematocrit level	LQC– 0.35 ng/mL			HQC– 45 ng/mL		
	0.24	0.43	0.64	0.24	0.43	0.64
Recovery [%]	96.78 ± 3.23	101.11 ± 1.41	103.45 ± 1.06	98.98 ± 4.06	100.87 ± 2.03	98.96 ± 1.98

### Cross-validation and clinical application

**Fig. 1:**
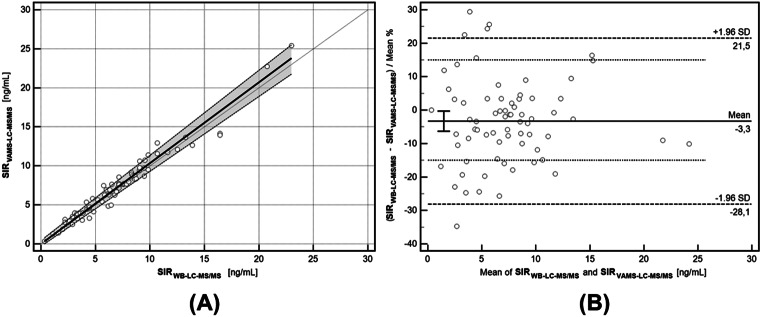
Passing-Bablok regression (**A**) and Bland-Altman bias estimation (**B**) for paired samples (description on the axis). Data are presented as empty circles for all paired samples. The regression line and confidence interval curve are presented as bold line and grey area, respectively, on the Passing-Bablok regression curve. The limit of agreement, average difference (also expressed as a percentage value), and ± 15% IATDMCT agreement limit are presented as ± 1.96 SD, solid and dotted lines, respectively. IATDMCT– International Association of Therapeutic Drug Monitoring and Clinical Toxicology

Clinical samples obtained from pediatric renal transplant patients during regular follow-up visits were analysed for SIR levels using the validated LC-MS/MS methods. A summary of the results obtained using the both validated methods is presented in the Supplementary File (part 4). The results for each group displayed a normal distribution, which was tested using the Shapiro-Wilk method. Additional clinical and cross-validation data are presented in Table 6; Fig. 1.

**Table 6:** Summary of statistics in cross- and clinical validation between SIR concentrations obtained using WB-LC-MS/MS and VAMS-LC-MS/MS methods. EMA- European Medicines Agency; IATDMCT- International Association of Therapeutic Monitoring and Clinical Toxicology; LC-MS/MS, liquid chromatography-tandem mass spectrometry; SIR_WB_, sirolimus concentration determined in whole blood sample using LC-MS/MS; SIR_VAMS_- Sirolimus concentration determined in VAMS sample using LC-MS/MS. data are presented as the mean with range and confidence intervals (95% CI)

Statistics	WB-LC-MS/MS versus VAMS-LC-MS/MS
Passing-Bablok regression formula	**SIR**_**VAMS**_ = 1.01(**SIR**_**WB**_) + 0.18
Intercept (A)	0.18 (-0.07 to 0.50); **0 within range**
Slope (B)	1.01 (0.95 to 1.08); **1 within range**
Mean Bland-Altman %-bias	-3.30%
% of paired samples fulfilled EMA criteria (< 20%)	89.33% **(> 67%; fulfilled)**
% of paired samples fulfilled IATDMCT criteria (< 15%)	76.00% **(> 67%; fulfilled)**
Correlation Pearson’s coefficient (R^2^)	0.9892 (0.9664–0.9972)
Spearman rank correlation coefficient	0.9820 (0.9713–0.9890)
Intraclass correlation coefficient	0.9893 (0.9826–0.9934)

**Fig. 2:**
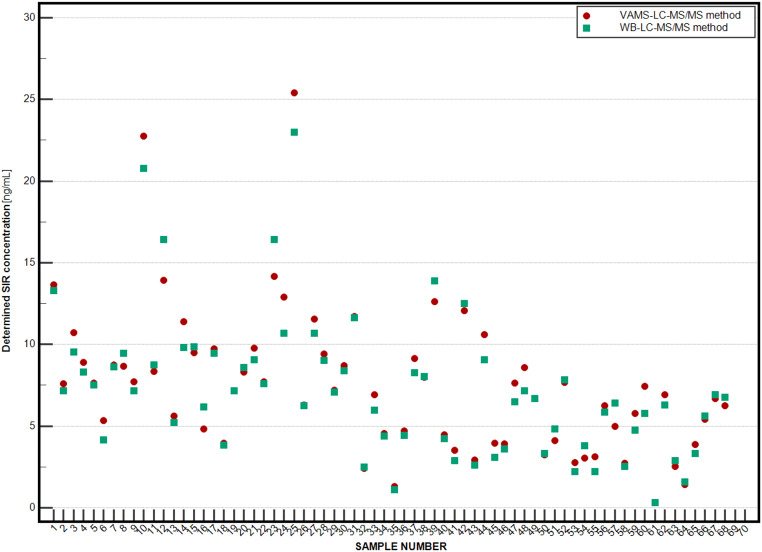
Comparison of sirolimus determination values using VAMS-LC-MS/MS and WB-LC-MS/MS assays for 70 sample sets (set is considered as whole blood sample and VAMS sample) obtained from pediatric renal transplant recipients [*n* = 25]. Mean (with SD and min-max range) SIR level measured in WB-LC-MS/MS method [ng/mL]: 7.11 ± 4.26 (0.32–22.98), and in VAMS-LC-MS/MS method [ng/mL]: 7.33 ± 4.41 (0.32–25.42). The legend presents the meaning of using points, shapes, and colours. The sample set was blinded using numbered symbols. LC-MS/MS, liquid chromatography-tandem mass spectrometry; WB, whole blood; VAMS, volumetric absorptive microsampling

The summarized results from the measurements divided by the analytical method type are shown in Fig. 2. The mean SIR levels.

Methods have been evaluated using a preliminary external international proficiency testing scheme. The initial results confirmed the operational performance of the validated methods. However, external proficiency testing should be performed systematically, especially for routinely used in clinical practice bioanalytical methods (Supplementary file - part 5).

## Discussion

This study introduced a novel approach for determining the SIR in venous and capillary whole blood samples obtained using a VAMS Mitra™ microsampling device. Our primary objective during the development of the method was to optimize the sample preparation protocol, chromatographic and detection conditions, and method validation in a lower concentration range of the SIR. The following outcomes were fully validated following the EMA guidelines on bioanalytical method validation and the IATDM&CT consensus [14, 16–18].

The method’s reliability was demonstrated through cross- and clinical validation using statistical tools such as the Passing–Bablok regression and Bland–Altman bias estimation, which showed good agreement between SIR concentrations determined in paired samples (WB versus VAMS). Although there are a few contributions to the scientific literature regarding microsampling implementation for SIR measurement [22–25], our study is the first to assess VAMS in TDM of the SIR in the pediatric population. During the pre-analytical phase, the sampling process is critical, regardless of the collection method used for the blood (or another matrix). For the pediatric population, ethical and legal considerations necessitate a minimised volume of collected samples. Therefore, applying the microsampling method in this population is strictly beneficial.

We employed a 10 µL type of Mitra™ sampler tip for the VAMS-LC-MS/MS method, representing a 120-fold reduction in volume compared to the routinely collected venous blood samples for TDM in our transplant centre. In published studies on SIR, 10 µL or 20 µL samplers were used, and after collecting the samples, they were stored under ambient conditions, without desiccant, or frozen at -20 °C [22–25]. To improve the sample stability, we dried the collected samples under ambient conditions for a minimum of 2 h before storing them in the dark at ambient temperature with desiccant (RT; 20 ± 8 °C; humidity < 45%). Although this is the shortest drying time reported, it was supported by the acceptable recovery during the extraction process. Several previously published studies have indicated that reducing the drying time to 2 h does not result in significant differences in recovery [5, 7].

Optimization of the sample preparation protocol is essential for appropriate purification, primarily when LC-MS/MS techniques are used. The dried capillary whole blood in the VAMS tip must be reconstituted using a suitable solvent; the proposed methodologies involve using methanol and/or water with sonication and/or vortexing [5, 7, 22–25]. Our protocol is based on an environmentally friendly extraction method using water assisted by short sonication and shaking. Biological samples can be purified using different methods, such as protein precipitation (PPT) or liquid-liquid extraction (LLE). In presented study, modified LLE technique, namely DLLME (dispersive liquid-liquid microextraction) was used. DLLME lean against the aqueous (containing analyte), organic and disperser (acetone, methanol, acetonitrile) solvent compositions. In fact, the relatively low amount of aqueous sample volume is enriched with a rapidly injected organic/disperser mixture. The advantages of this type of extraction, compared to standard LLE, is a faster extraction process with a higher analyte recovery rate (i.e. due to appreciable contact surface area between organic and aqueous phases, additionally increased by disperser presence in sample), as well as the possibility of organic solvent volume reduction [26–27]. In our protocol, extraction with water resulted in a 150 µL extract, but two-step purification resulted in a concentrated sample. The technique is more environmentally friendly than LLE– an appropriate evaluation of developed methods is provided in the Supplemental material (part 6). The AGREE scale was used for the green character of analytical procedures assessment (0.59 and 0.65, respectively, for WB-LC-MS/MS and VAMS-LC-MS/MS methods) [28].

Subsequently, only 5 µL of supernatant can be injected into the LC-MS/MS system, whereas other studies have used volumes ranging from 10 to 40 µL [22–25]. Notably, our optimised protocol for VAMS sample treatment was the first to use the DLLME technique, but similar approaches have been reported for WB-LC-MS/MS methods [27, 28].

Determination of immunosuppressive drugs using LC-MS/MS is considered the gold standard for TDM. However, it is the sole method for determining these drugs in small blood volumes collected using microsampling techniques such as VAMS. Our study employed an ESI(+)-LC-MS/MS system, which utilises chromatographic separation with an octadecyl phase. The total run time of 4.50 min was optimised to ensure optimal column washing and system equilibration. Theoretically, the VAMS method presented in this study can process over 300 samples per 24 h, resulting in a high processing capacity and robustness (run time for SIR-VAMS published methods ranged from 2.2 min to 3.0 min) [5, 22–25].

The current study aimed to develop two reliable cross-and clinical validation methods: reference WB-LC-MS/MS and VAMS-LC-MS/MS. In the reference method, three internal standards, ASC, SIR-d_3_, and TEMS, were used. Notably, ASC was utilized for VAMS-based method calibration because of its acceptable method performance and cost-effectiveness. In contrast, TEMS has been used in other studies as an internal standard. Still, our method exhibited limited recovery (ranging from 55 to 67%, depending on the concentration) and limited stability during sample storage in an autosampler [23]. Due to its attractive cost, only ASC was used for VAMS-LC-MS/MS method validation because of its superior stability to SIR-d_3_ and TEMS (one-year stability of the working solution was higher for the ASC than for the SIR-d_3_).

The stability of the VAMS tips was evaluated for one month under various conditions, and it was found that the SIR showed adequate stability at -20 °C, RT (in the dark), and 4 °C. However, acceptable stability was observed for only 3 days at 60 °C. Previous studies by Koster et al. and Paniagua-Gonzalez et al. reported different stability findings for VAMS under various conditions, including 50 days at -20 °C and 2 days at 50 °C for SIR stability, and 8 months at -20 °C and 15 days at 4 °C and 20 °C for analyte stability [22, 23]. The stability after sampling should be evaluated under different conditions according to the logistic process of obtaining samples from TDM laboratories using the classic post. The VAMS-Mitra™ device theoretically presents hematocrit independence in conformance with recovery, and the hematocrit effect was evaluated in this study, similar to previously published protocols. No hematocrit effect was observed in a vast percentage of the hematocrit (14.3–64.1%) during recovery rates analysis (Table 5), consistent with previous reports. It should be noted that a correlation between SIR concentration differences (the WB and VAMS samples) and individual hematocrit levels (ranging from 28.3 to 47.3%) for each sample set was also observed, and the relationship between mentioned variables was characterized by weak correlation significance.

As our study presented the clinical application of the microsampling technique, where samples were collected by medical doctors, nurses, patients, and their family members after training in the hospital, we did not observe any errors in the sampling process [5, 18].

One of the strengths of our study was our participation in the preliminary rounds of the LGC external proficiency testing scheme, which confirmed the agreement of our methods with satisfactory results. Both WB-LC-MS/MS and VAMS-LC-MS/MS methods yielded acceptable results. On the other hand, a limitation of our study is that it did not include blood collection at home. Additionally, some validation tests were not performed, i.e. injection sol-vent stability assessment, dilution integrity and extended stability test, which might be considered a limitation. Nevertheless, the stability examination results indicated that the samples could be transported via post. The key to success in terms of VAMS sample quality is proper training of the patient and their family in home self-sampling. One of the critical points of sampling is adequate quality assurance regarding sampling correctness and analyte stability. In the case of capillary whole blood collecting, the VAMS sample is correctly loaded when the tip is entirely red. Therefore, sample quality is easy for patients and medical personnel to evaluate. Since SIR stability studies in capillary whole blood collected using the VAMS technique have been limited to specific conditions in the presented research (-20 °C, 4 °C, RT and 60 °C; humidity < 45%), patients should be advised on adequate handling the sample after collection. The sample should be dried under ambient conditions for 2 h in the cartridge or clamshell provided by the manufacturer after collection. This protects from light and adequate drying conditions. The whole should be placed in an aluminium zip bag with a moisture absorber. Based on the stability studies performed, sending the sample under these conditions (without significant deviations from ambient temperature) ensures adequate SIR stability. There is no doubt, however, that extended stability studies simulating various sample storage and shipping conditions should be performed before the final introduction of this technique for remote monitoring of immunosuppressive therapy using SIR.

## Conclusions

The WB-LC-MS/MS and VAMS-LC-MS/MS methods were successfully developed and validated. Furthermore, the clinical equivalence between the reference (WB-LC-MS/MS) and microsampling (VAMS-LC-MS/MS) methods was established. In contrast to the routinely used methods (IAs and LC-MS/MS assays using WB), microsampling presents additional challenges for TDM laboratories, medical practitioners, and diagnosticians. However, it may also benefit patients, their families, and the healthcare system. Because of its wide calibration range, the developed and validated method can be employed in pharmacokinetic studies and clinical trials. The evaluated sample stability and pilot patient training during follow-up visits will allow for home-based self-sampling and shipping of the samples to the TDM laboratory. To the best of our knowledge, the presented study is the first application of the VAMS technique for therapeutic drug monitoring of SIR during immunosuppressive pharmacotherapy in a pediatric population.

## Electronic supplementary material

Below is the link to the electronic supplementary material.

Supplementary Material 1

## Data Availability

The basic data supporting the findings of this study are available in the paper and its Supplementary Information. Detailed references to the individual supplementary file are provided in the manuscript. Other datasets generated during and/or analysed during the current study are available from the corresponding author upon reasonable request.

## Abbreviations

ACMIA: Antibody Conjugated Magnetic Immunoassay
AR: Absolute recovery
ASC: Ascomycin
C_0_: Trough concentration
CE: Collision energy
CMIA: Chemiluminescent Microparticle Immunoassay
CS: Calibration standard
CV: Coefficient of variation
DLLME: Dispersive liquid-liquid microextraction
EMA: European Medicines Agency
EMIT: Enzyme Multiplied Immunoassay Technique
F: IS normalized matrix factor
FDA: Food and Drug Administration
HQC: Highest quality control
IAs: Immunochemical assays
IATDM&CT: International Association of Therapeutic Drug Monitoring and Clinical Toxicology
IS: Standard
ISR: Incurred sample reanalysis
LC-MS/MS: Liquid chromatography-tandem mass spectrometry
LLOQ: Lower limit of quantification
LOD: Limit of detection
LQC: Lower quality control
ME: Matrix effect
MQC: Medium quality control
MRM: Multiple reaction monitoring
MTBE: Methyl tert-butyl ether
PE: Process efficacy
PPT: Protein precipitation
QC: Quality control
RT: Room temperature
S/N: Signal to noise
SD: Standard deviation
SIL-IS: Stable isotope-labelled internal standard
SIR: Sirolimus
SIR-d_3_: Deuterated sirolimus
qDBS: Quantitative dried blood spot
TDM: Therapeutic Drug Monitoring
TEMS: Temsirolimus
VAMS: Volumetric Absorptive Microsampling
WB: Whole blood
