# Supplementary material for: Personalization of pharmacotherapy with sirolimus based on volumetric absorptive microsampling (VAMS) in pediatric renal transplant recipients—from LC-MS/MS method validation to clinical application
Source: Pharmacol Rep. 2024 Oct 10;76(6):1443–55. doi: 10.1007/s43440-024-00663-9 (PMC11582253; doi:10.1007/s43440-024-00663-9)
Supplement: Supplementary file 1 — Supplementary Material 1 [file 43440_2024_663_MOESM1_ESM.docx]

**SUPPLEMENTARY FILE**

**Personalization of Pharmacotherapy with Sirolimus based on Volumetric Absorptive Microsampling (VAMS) in Pediatric Renal Transplant Recipients—from LC-MS/MS Method Validation to Clinical Application**

Arkadiusz Kocur^1,2,*^ (AK); Agnieszka Czajkowska^2^ (AC); Kamila Rębis^3^ (KR); Jacek Rubik^4^ (JR); Mateusz Moczulski^3^ (MM); Bartłomiej Kot^3^ (BK); Maciej Sierakowski^5^ (MS); Tomasz Pawiński^1^ (TP)

^1^Department of Drug Chemistry, Pharmaceutical and Biomedical Analysis, Faculty of Pharmacy, Medical University of Warsaw, Banacha 1, 02-097 Warsaw, Poland. ORCID: 0000-0002-4833-8532 (AK); 0000-0001-9110-4312 (TP).

^2^Therapeutic Drug Monitoring, Clinical Pharmacokinetics and Toxicology Laboratory Unit, Department of Clinical Biochemistry, The Children’s Memorial Health Institute, Dzieci Polskich 20, 04-730 Warsaw, Poland. ORCID: 0000-0003-0199-5751 (AC).

^3^Students Scientific Association “Drug” in the Department of Drug Chemistry, Pharmaceutical and Biomedical Analysis, Faculty of Pharmacy, Medical University of Warsaw, Banacha 1, 02-097 Warsaw, Poland.

^4^Department of Nephrology, Kidney Transplantation and Arterial Hypertension, The Children’s Memorial Health Institute, Dzieci Polskich 20, 04-730 Warsaw, Poland. ORCID: 0000-0002-3392-2154 (JR).

^5^Institute of Biological Sciences, Cardinal Stefan Wyszynski University, Kazimierza Wóycickiego 1/3, 01-938, Warsaw, Poland. ORCID: 0000-0003-3770-2762.

* Correspondence: Arkadiusz Kocur, Department of Drug Chemistry, Pharmaceutical and Biomedical Analysis, Faculty of Pharmacy, Medical University of Warsaw, 02-097 Warsaw, Poland. Tel.: (+48 22) 57 20 630; Fax.: (+48 22) 57 20 697; E-mail address: arkadiusz.kocur@wum.edu.pl

** Mateusz Moczulski and Bartłomiej Kot contributed equally to this work.

**Running head:** Personalized Sirolimus therapy in pediatric population.

**Part 1.**

**Working solution preparation protocol**

**Stock solution:** 1 mg/mL (1000 µg/mL) (prepared twice = two independent amount of solid SIR standard dissolved in MeOH: water mixture, 1:1 (v/v) ad 10 mL)

**‘’Auxiliary’’ intermediate solutions:** 0.1 mg/mL (100 µg/mL) (prepared twice)

**Working solution:** (prepared twice = separated for CC and QC preparation)

A1a: 10000 ng/mL (10 µg/mL)

A1b: 10000 ng/mL (10 µg/mL)

A2a: 1000 ng/mL (1 µg/mL)

A2b: 1000 ng/mL (1 µg/mL)

A3a: 100 ng/mL (0.1 µg/mL)

A3b: 100 ng/mL (0.1 µg/mL)

| CC | QC | Final nominal concentration of SIR in prepared CC or QC | Working CC/QC solutions corresponding with final CC/QC were prepared using pure deionized water (ad 2 mL) |
| --- | --- | --- | --- |
| 1  LLOQ | **-** | **0.25** | **25 µL of A3a** (water ad 1975 µL) |
| 2 | **-** | **0.50** | **50 µL of A3a** (water ad 1950 µL) |
| 3 | **-** | **1.00** | **100 µL of A3a** (water ad 1900 µL) |
| 4 | **-** | **2.50** | **25 µL of A2a** (water ad 1975 µL) |
| 5 | **-** | **5.00** | **50 µL of A2a** (water ad 1950 µL) |
| 6 | **-** | **10.00** | **100 µL of A2a** (water ad 1900 µL) |
| 7 | **-** | **30.00** | **30 µL of A1a** (water ad 1970 µL) |
| 8  ULOQ | **-** | **60.00** | **60 µL of A1a** (water ad 1940 µL) |
| - | **A**  LQC | **0.35** | **35 µL of A3b** (water ad 1965 µL) |
| - | **B**  MQC1 | **0.75** | **75 µL of A3b** (water ad 1925 µL) |
| - | **C**  MQC2 | **3.50** | **35 µL of A2b** (water ad 1965 µL) |
| - | **D**  MQC3 | **25.00** | **25 µL of A1b** (water ad 1975 µL) |
| - | **E**  HQC | **45.00** | **45 µL of A1b** (water ad 1955 µL) |

- CS – calibration standard; QC – quality control
- LLOQ – lower limit of quantification, ULOQ – upper limit of quantification
- LQC – lower quality control, MQC1 – 1^st^ medium quality control, MQC2 – 2^nd^ medium quality control, MQC3 – 3rd medium quality control, HQC – higher quality control

**Part 2. Representative chromatograms**

| 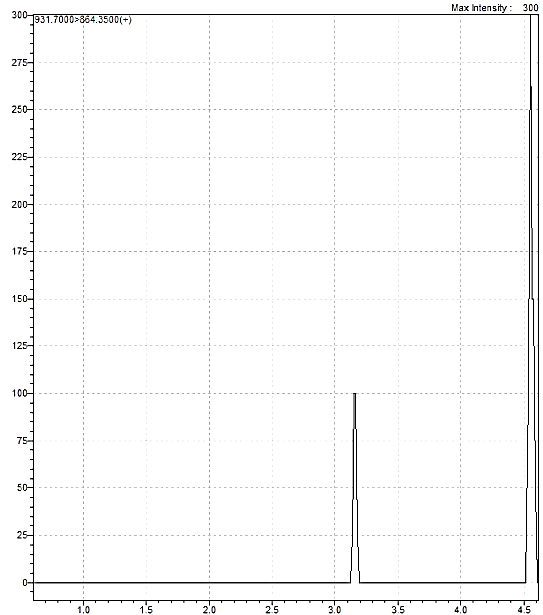 | 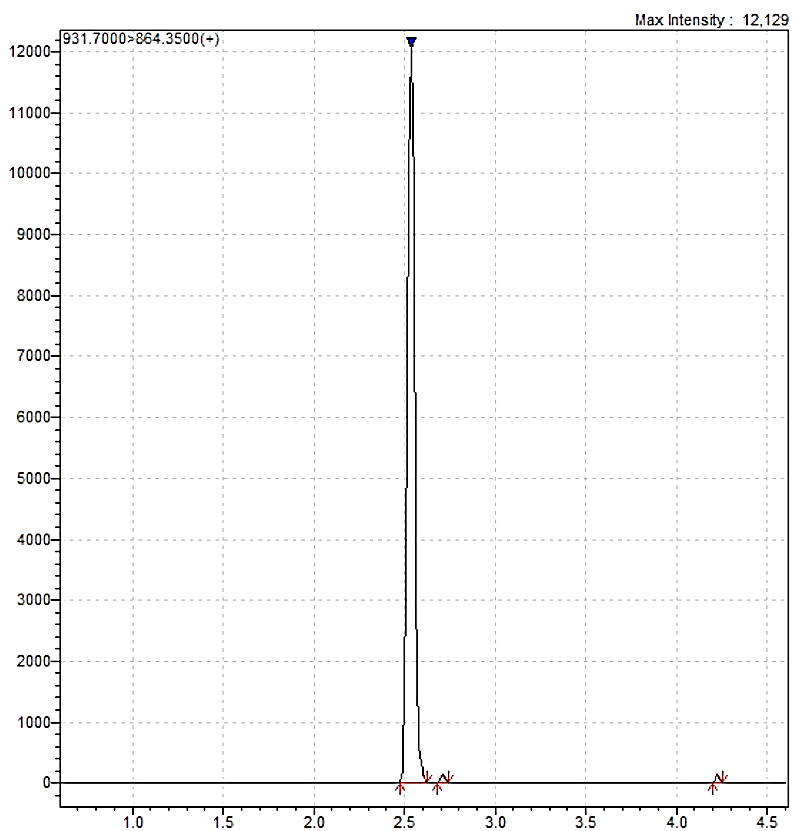 |
| --- | --- |
| A | B |
| 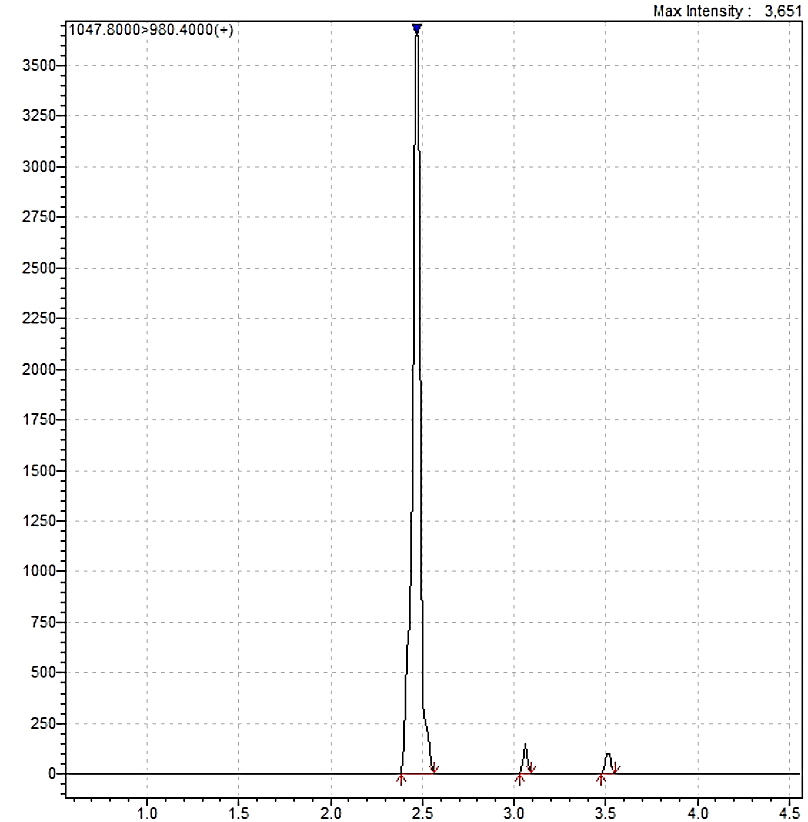 | 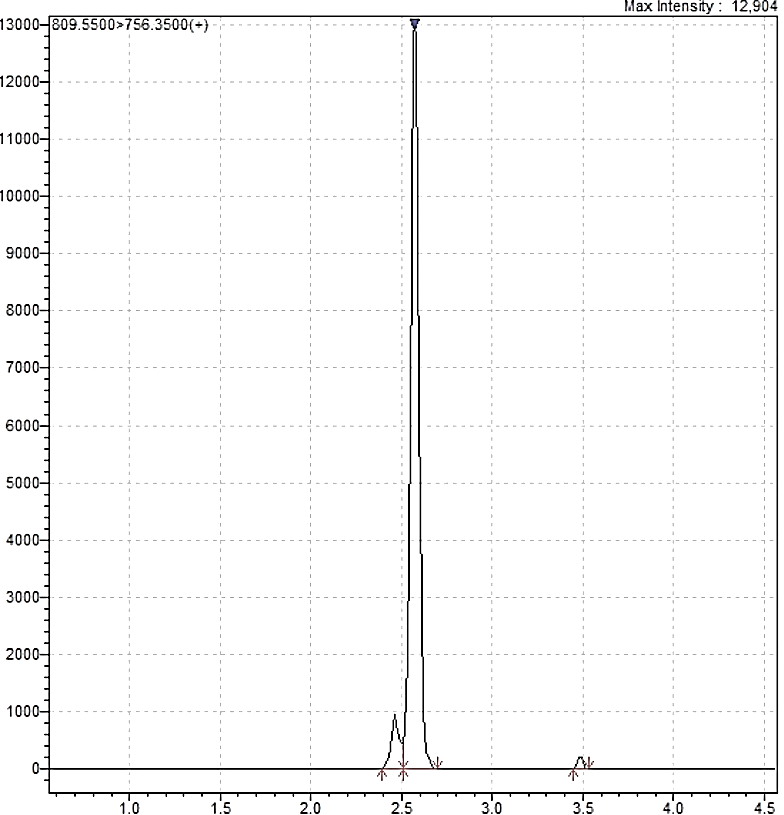 |
| C | D |
| 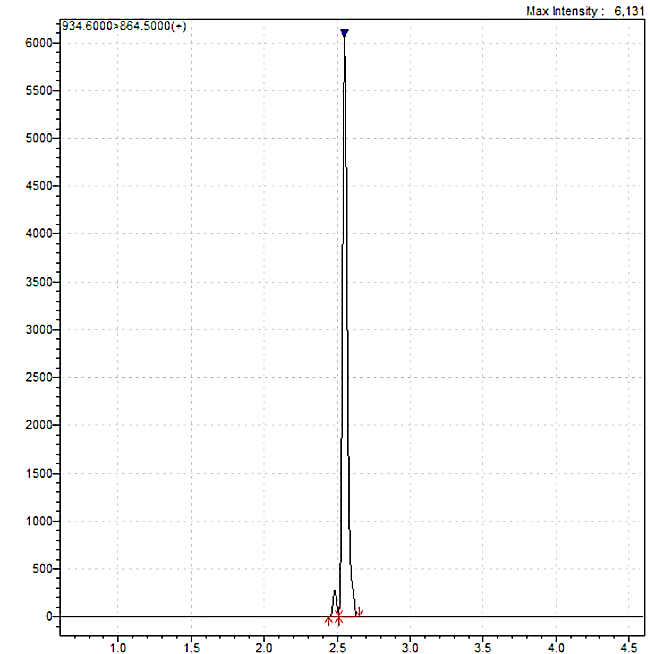 | |
| E | |

**Figure 1.** Representative chromatograms of: (A) blank sample, (B) sirolimus, (C) IS: temsirolimus, (D) IS: ascomycin, (E) IS: sirolimus-d3. IS – internal standard.

**Part 3. Result of WB-LC-MS/MS method validation for SIR using three ISs (SIR-d_3_, ASC, TEMS)**

**Table 1.** Accuracy and precision parameters based on within-day (intra-day) and between-day (inter-day) repetition analysis for the WB-LC-MS/MS method [n=10]. ASC, ascomycin; LOQ, lower quality control; MQC_1_ – 1^st^ medium quality control; MQC_2_ – 2^nd^ medium quality control; MQC_3_ – 3^rd^ medium quality control; HQC, higher quality control; SIR-d_3_, deuterated sirolimus; TEMS, temsirolimus.

| **INTRA-DAY** | LOQ  0.35 ng/mL | MQC_1_  0.75 ng/mL | MQC_2_  3.50 ng/mL | MQC_3_  25 ng/mL | HQC  45 ng/mL |
| --- | --- | --- | --- | --- | --- |
| **IS: ASC** | | | | | |
| Mean concentration  [ng/mL] | 0.36±0.02 | 0.78±0.04 | 3.67±0.15 | 25.70±0.54 | 45.49±0.54 |
| Accuracy [%] | 106.61 | 101.53 | 105.16 | 99.93 | 100.15 |
| Precision [%] | 6.53 | 4.52 | 4.19 | 2.11 | 1.18 |
| **IS: SIR-d_3_** | | | | | |
| Mean concentration  [ng/mL] | 0.36±0.02 | 0.76±0.04 | 3.51±0.16 | 25.76±0.92 | 45.46±0.97 |
| Accuracy [%] | 91.78 | 99.58 | 101.20 | 100.18 | 100.07 |
| Precision [%] | 6.95 | 5.18 | 4.61 | 3.54 | 2.14 |
| **IS: TEMS** | | | | | |
| Mean concentration  [ng/mL] | 0.35±0.04 | 0.78±0.05 | 3.49±0.22 | 24.87±0.88 | 44.70±1.12 |
| Accuracy [%] | 88.76 | 100.84 | 99.79 | 96.74 | 98.39 |
| Precision [%] | 10.38 | 5.97 | 6.31 | 3.53 | 2.51 |

| **INTER-DAY** | LOQ  0.35 ng/mL | MQC_1_  0.75 ng/mL | MQC_2_  3.50 ng/mL | MQC_3_  25 ng/mL | HQC  45 ng/mL |
| --- | --- | --- | --- | --- | --- |
| **IS: ASC** | | | | | |
| Mean concentration  [ng/mL] | 0.36±0.01 | 0.76±0.02 | 3.50±0.08 | 25.12±0.54 | 45.44±0.46 |
| Accuracy [%] | 95.11 | 100.26 | 100.18 | 100.38 | 100.08 |
| Precision [%] | 3.77 | 2.44 | 2.37 | 2.18 | 1.00 |
| **IS: SIR-d_3_** | | | | | |
| Mean concentration  [ng/mL] | 0.36±0.02 | 0.75±0.03 | 3.59±0.08 | 25.70±0.53 | 45.25±0.69 |
| Accuracy [%] | 99.05 | 99.95 | 99.09 | 102.73 | 99.69 |
| Precision [%] | 4.32 | 3.56 | 2.39 | 2.09 | 1.53 |
| **IS: TEMS** | | | | | |
| Mean concentration  [ng/mL] | 0.37±0.02 | 0.76±0.06 | 3.56±0.15 | 24.99±1.01 | 45.07±0.77 |
| Accuracy [%] | 103.59 | 100.69 | 100.49 | 99.90 | 99.26 |
| Precision [%] | 5.40 | 4.87 | 4.16 | 4.07 | 1.78 |

Data are presented as mean with standard deviation (mean ± SD).

**Table 2.** Stability evaluation in the autosampler for the WB-LC-MS/MS method [n=6]. ASC, ascomycin; LOQ, lower quality control; HQC, higher quality control; SIR-d_3_, deuterated sirolimus; TEMS, temsirolimus.

| Calculated Concentration [ng/mL] and Stability [%] | | | | | |
| --- | --- | --- | --- | --- | --- |
|  | Initial | Day 1 | Day 3 | Day 5 | Day 7 |
| **IS: ASC** | | | | | |
| LQC – 0.35 ng/mL | 0.33±0.09;  100% | 0.34±0.11;  103.03% | 0.32±0.06;  96.97% | 0.31±0.12;  93.94% | 0.28±0.09;  85.84% |
| HQC – 45 ng/mL | 45.26±1.16; 100% | 45.13±1.53; 99.71% | 44.89±1.10; 99.18% | 44.31±1.67; 97.90% | 43.89±1.98; 96.97% |
| **IS: SIR-d_3_** | | | | | |
| LQC – 0.35 ng/mL | 0.34±0.13;  100% | 0.31±0.21;  91.18% | 0.32±0.19;  94.12% | 0.29±0.09;  85.29% | 0.27±0.14;  79.41% |
| HQC – 45 ng/mL | 45.73±1.96; 100% | 44.98±2.12; 98.36% | 44.65±2.61; 97.64% | 43.33±2.89; 94.75% | 42.05±3.01; 91.95% |
| **IS: TEMS** | | | | | |
| LQC – 0.35 ng/mL | 0.36±0.13;  100% | 0.35±0.19;  97.22% | 0.31±0.17;  86.11% | 0.26±0.20;  72.22% | 0.24±0.19;  66.67% |
| HQC – 45 ng/mL | 46.28±3.67; 100% | 45.22±3.23; 97.71% | 43.79±2.83; 94.62% | 41.92±2.75; 90.58% | 40.45±3.35; 84.40% |

Data are presented as mean with standard deviation (mean ± SD).

**Table 3.** Matrix effect (ME), absolute recovery (AR), and process efficiency (PE) data for WB-LC-MS/MS using three IS [n=6]. ASC, ascomycin; LOQ, lower quality control; HQC, higher quality control; SIR-d_3_, deuterated sirolimus; TEMS, temsirolimus.

| **LQC – 0.35 ng/mL** | | | | | | | |
| --- | --- | --- | --- | --- | --- | --- | --- |
| Parameter | SIR | IS-ASC | IS-SIR-d_3_ | IS-TEMS | F  (SIR/ASC) | F  (SIR/ SIR-d_3)_ | F (SIR/TEMS) |
| ME [%] | −22.89 ± 9.34 | −33.15± 8.51 | −39.22 ± 6.24 | −35.29 ± 7.33 | 88.06 ± 11.54 | 91.68 ± 19.01 | 79.94 ± 21.66 |
| PE [%] | 76.22 ± 3.44 | 69.69 ± 4.54 | 64.19 ± 2.99 | 68.21 ± 3.56 | 101.13 ± 2.44 | 98.47 ± 4.40 | 97.53 ± 2.18 |
| AR [%] | 73.08 ± 2.85 | 61.13 ± 3.57 | 60.07 ± 4.01 | 58.09 ± 2.99 | 99.31 ± 2.18 | 99.82 ± 2.91 | 103.22 ± 4.21 |
| **HQC – 45 ng/mL** | | | | | | | |
| ME [%] | −24.64 ± 5.43 | −30.15± 4.99 | −29.86 ± 4.51 | −30.49 ± 5.22 | 94.26 ± 14.77 | 97.06 ± 9.33 | 88.78 ± 15.43 |
| PE [%] | 71.39 ± 3.84 | 64.21 ± 3.67 | 68.41 ± 3.89 | 63.26 ± 4.18 | 99.41 ± 2.10 | 102.77 ± 3.66 | 100.03 ± 8.43 |
| AR [%] | 75.13 ± 2.99 | 67.33 ± 3.78 | 65.77 ± 2.99 | 68.29 ± 4.59 | 96.66 ± 3.47 | 98.88 ± 4.02 | 95.32 ± 3.99 |

Data are presented as mean with standard deviation (mean ± SD).

**Part 4. Clinical results of SIR determination in samples from renal pediatric transplant recipients received SIR.**

**Table 4.** Results of clinical samples determination using WB-LC-MS/MS, VAMS-LC-MS/MS methods. WB-LC-MS/MS – liquid chromatography – tandem mass spectrometry method sirolimus determination in whole blood; VAMS-LC-MS/MS – liquid chromatography – tandem mass spectrometry method sirolimus determination in VAMS sample; VAMS – volumetric-absorptive microsampling.

Samples set WB-LC-MS/MS VAMS-LC-MS/MS

[ng/mL] [ng/mL]


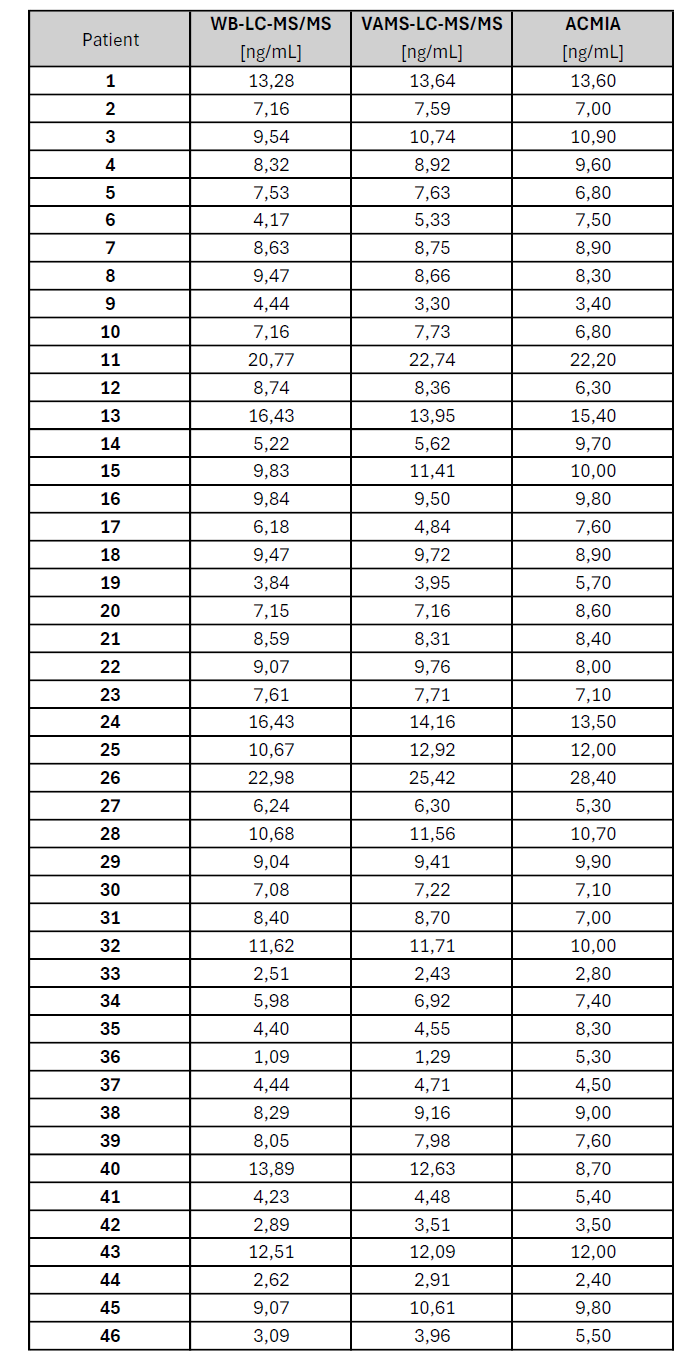


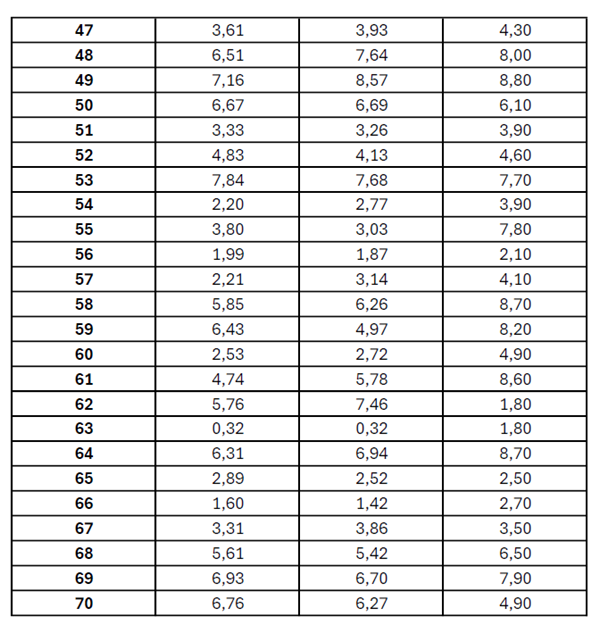


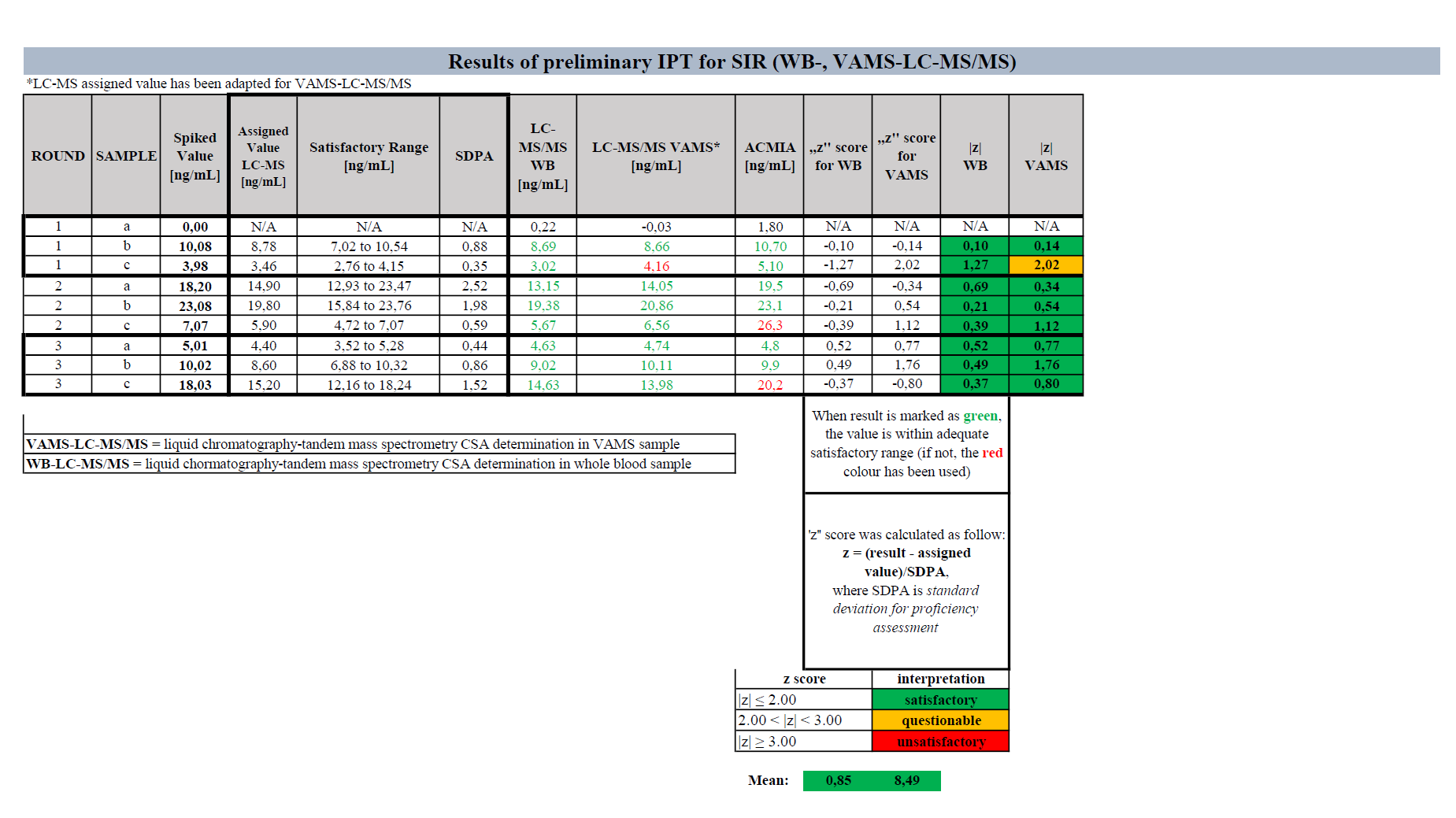
**Part 5.** **Results of external international proficiency testing (IPT) for sirolimus**

**Table 5.** Results of external international proficiency testing (IPT) for sirolimus measured with assays: WB-LC-MS/MS and VAMS-LC-MS/MS. SDPA – standard deviation for proficiency assessment, WB-LC-MS/MS (liquid chromatography – tandem mass spectrometry method sirolimus determination in whole blood), VAMS-LC-MS/MS (liquid chromatography – tandem mass spectrometry method sirolimus determination in whole blood).

**Part 6. Comparison of greenness of validated methods** (The AGREE scale for analytycial method assessment according to analytical procedure’s greenness).

Na podstawie: Pena-Pereira F, Wojnowski W, Tobiszewski M. AGREE-Analytical GREEnness Metric Approach and Software. Anal Chem. 2020 Jul 21;92(14):10076-10082. doi: 10.1021/acs.analchem.0c01887.


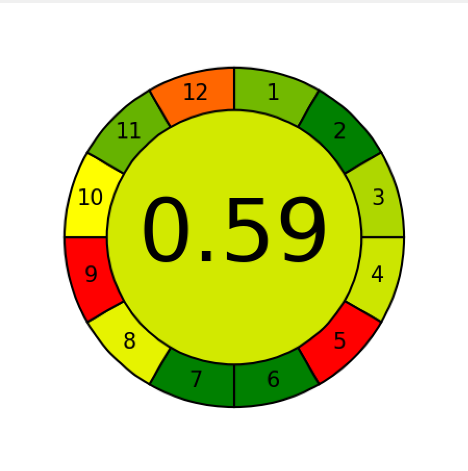


1.
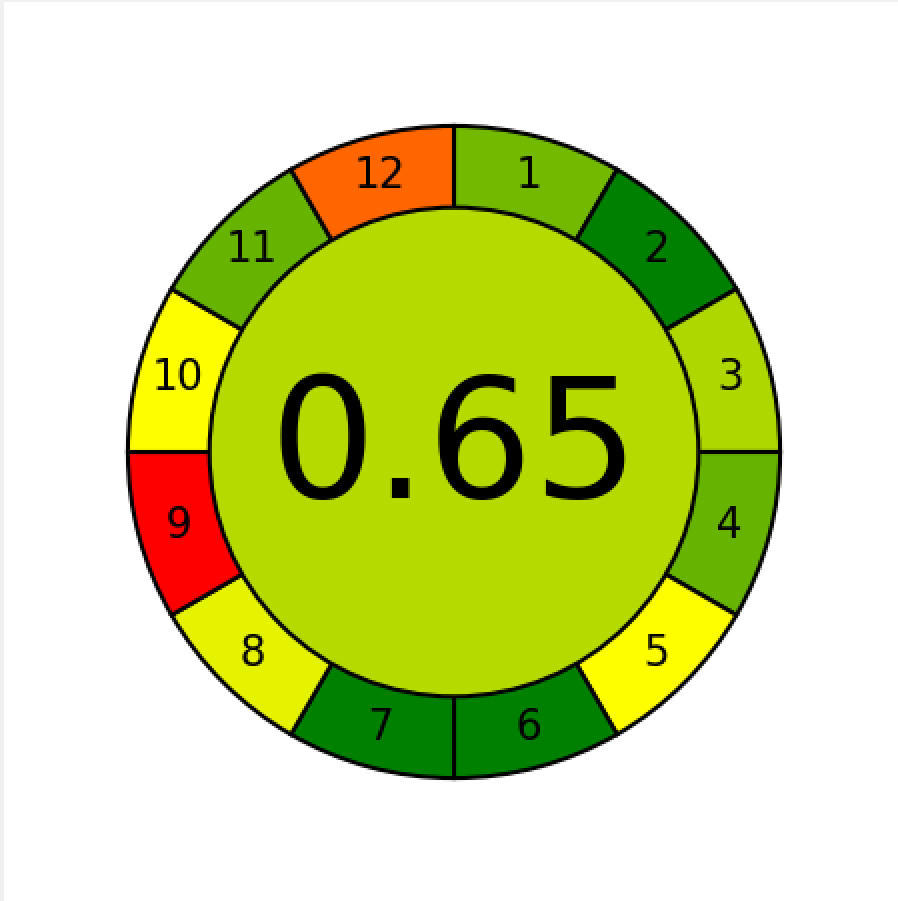
 (B)

**Figure 2.** Results of AGREE analysis for (A) WB-LC-MS/MS and (B) VAMS-LC-MS/MS methods. WB-LC-MS/MS (liquid chromatography – tandem mass spectrometry method sirolimus determination in whole blood), VAMS-LC-MS/MS (liquid chromatography – tandem mass spectrometry method sirolimus determination in whole blood). Scale description: 10.1021/acs.analchem.0c01887.
